# No evidence of tocilizumab treatment efficacy for severe to critical SARS-CoV2 infected patients: Results from a retrospective controlled multicenter study

**DOI:** 10.1097/MD.0000000000026023

**Published:** 2021-05-28

**Authors:** Ruxandra Burlacu, Jonathan London, Audrey Fleury, Thomas Sené, Abdourahmane Diallo, Vanina Meyssonnier, Valérie Zeller, Joris Galland, Tessa Huscenot, Emma Rubenstein, Pierre Trouiller, Roland Amathieu, Johannes Kutter, David Blondeel, Gabriel Lejour, Stéphane Mouly, Olivier Lidove, Mauhin Wladimir, Damien Sène

**Affiliations:** a Department of Internal Medicine, Lariboisière Fernand Widal Hospital, AP-HP, University of Paris; b Department of Internal Medicine and Infectious Diseases, Diaconesses Croix-Saint Simon Hospital; c Reproductive Medicine Unit, Diaconesses Croix-Saint Simon Hospital; d Department of Internal Medicine, Rothschild Hospital Foundation; e Biostatistics and Clinical Trial Unit, Lariboisière Fernand Widal Hospital, AP-HP; f Intensive Care Unit, Rothschild Hospital Foundation; g Intensive Care Unit, Diaconesses Croix-Saint Simon Hospital; h Anesthesiology Department, Diaconesses Croix-Saint Simon Hospital; i Emergency Department, Diaconesses Croix-Saint Simon Hospital, Paris, France.

**Keywords:** SARS-CoV2 infection, severe COVID-19, tocilizumab

## Abstract

To assess tocilizumab (TCZ) efficacy associated to standard of care (SOC) compared to SOC alone in severe coronavirus associated disease 2019 (COVID-19) patients. In a matched case-control study from 3 French Hospital COVID-19 Departments, 27 patients with severe COVID-19 treated with TCZ and SOC were matched for baseline epidemiological and clinical features and compared to 27 severe COVID-19 patients treated with SOC alone. Baseline characteristics of the study population were comparable between groups. Eleven patients (20%) died. TCZ was not associated with clinical improvement as compared to SOC regarding oxygen-free status (44% vs 63%) and death (18.5% vs 22%), despite a higher decrease of the C-reactive protein at Day 7 (10.7 vs 52 mg/L; *P* < 10^−3^). Compared to the 43 patients alive at the end-of follow-up, patients who died were older (78 vs 64 years; *P* < 10^−3^), with 82% of them older than 72 years vs only 23% of live patients (*P* < 10^−3^). Age (OR = 1.15; 95%CI = 1.04–1.3; *P* = .008) and age over 72 years (OR) = 14.85; 95%CI = 2.7–80; *P* = .002) were independently associated with mortality. TCZ in addition to SOC for severe COVID-19 patients did not reduce mortality, subsequent need for invasive mechanical ventilation nor did it shorten the time of oxygen support, despite better control of the inflammatory response. More powerful and randomized controlled trials are warranted to determine if TCZ is effective in the management of COVID-19.

## 1 Introduction

The emergent outbreak of coronavirus disease 2019 (COVID-19) caused by the severe acute respiratory syndrome coronavirus 2 (SARS-CoV-2) has resulted in a global pandemic.^[1]^ Approximately 25% to 30% of COVID-19 patients required hospitalization with severe complications including acute respiratory distress syndrome (ARDS) and intensive care unit (ICU) admission.^[2,3]^ Severe ARDS is the leading cause of death in COVID-19. There are currently no effective therapies to prevent severe forms of COVID-19 and no treatment has yet proven effective in reducing mortality in these patients.

Elevated plasmatic levels of inflammatory cytokines such as interleukin-6 (IL-6) and others, in COVID-19, also known as cytokine release syndrome (CRS) plays a key role in the pathology of severe COVID-19^[4–6]^ as suggested by the correlation between plasma IL-6 levels and COVID-19 severity.^[7,8]^ Before the demonstration of efficacy of corticosteroids in COVID-19 by the Recovery and Solidarity trials,^[9–11]^ there was an urgent need for novel therapies to treat COVID-19 associated/induced CRS.

Tocilizumab (TCZ) is an anti-human IL-6 receptor (IL-6R) monoclonal antibody that inhibits IL-6 signaling by binding soluble IL-6R and membrane IL-6R, blocking by this way its pro-inflammatory proprieties. TCZ is approved for rheumatoid arthritis, juvenile inflammatory arthritis and refractory giant cell arteritis, with a good tolerance except for the risk of bacterial infection and sigmoiditis. TCZ is also considered to be effective for the treatment of chimeric antigen receptor T cell therapy (CAR T-cells) related CRS.^[12]^ Considering the key role of IL-6 blocking by TCZ in the treatment of CAR T-cells CRS and the role of IL-6 in COVID-19 CRS, TCZ was rapidly considered as a potential benefit treatment for COVID-19 CRS. Preliminary published investigations (retrospective uncontrolled and case-controlled) reported unconclusive results.^[13–16]^ In July 2020, an online published metanalysis of 7 controlled studies showed no difference on death or ICU admission between TCZ treated groups and SOC groups^,^^[17]^ whereas 2 recent papers, published in August 2020, reported a reduced risk of invasive mechanical ventilation (IMV) and of death in TCZ treated patients when compared to those receiving SOC.^[18–20]^

We herein report the results of a retrospective matched case-control study looking at the efficacy of a TCZ treatment plus SOC compared to SOC alone in COVID-19 patients, on mortality, necessity of ICU admission and need for IMV. We also discuss the results of the available studies on the use of TCZ in severe COVID-19.

## 2 Patients and methods

### 2.1 Study population and design

We performed a multicenter retrospective analysis of all SARS-CoV-2 infected patients treated with TCZ (n = 27) in addition to SOC (TCZ group) from 3 French hospitals (Lariboisière APHP Hospital, Diaconesses Croix Saint-Simon Hospital, Rothschild Foundation Hospital) between March and April 2020. These patients were matched for baseline epidemiological (age, sex, hypertension, diabetes, obesity, chronic heart or lung disease) and clinical features (infection severity at admission, i.e. respiratory rate, oxygen saturation in room air) to 27 patients who received only SOC (non-TCZ group). Of note, most of the 27 patients included in the control group were admitted in March 2020, before we started to introduce TCZ for severe COVID-19.

All 54 included patients had clinical symptoms of respiratory infection and a positive nasopharyngeal swab polymerase chain reaction test for SARS-CoV-2 (Cobas SARS-CoV-2 – ROCHE). A chest Computed Tomography – scan was performed in 48 patients and was consistent with SARS-CoV-2 infection with variable degrees of lung involvement (<25%; 25%–50%; >50%). Epidemiological, clinical, laboratory and therapeutic data were recorded from patients’ medical records. These collected data are anonymized, stored and made available if necessary.

### 2.2 Treatment protocol

At the time the study was designed (between March and April 2020), the SOC for the treatment of COVID-19 patients in France consisted in oxygen therapy (adapted to oxygen saturation objectives), antibiotics (mostly ceftriaxone or cefotaxime plus azithromycin or rovamycin), antivirals (lopinavir/ritonavir; hydroxychloroquine) according to physician's decision and intensive life support (high flow, non-invasive ventilation, invasive ventilation, prone positioning) care when needed. All patients received anticoagulant treatment: standard prophylactic low molecular weight heparin for 22 patients, prophylactic body mass index-adapted dosing for eight patients and therapeutic dosing for 14 patients (one patient received apixaban). As their benefit in COVID-19 management was debatable, only 14 patients (26%) received steroids treatment.

TCZ was compassionately administered to 27 patients with severe COVID-19, following local guidelines and ethical procedures, after multidisciplinary concertation. Severe COVID-19 was diagnosed in patients requiring at least 6 L/min of oxygen flow to reach a SpO_2_ ≥ 94%, 7 to 15 days after symptoms onset. Confirmed bacterial or fungal co-infection before TCZ were exclusion criteria. Twenty-four patients received 1 dose of intravenous TCZ (8 mg/kg) and 3 patients received 1 subcutaneous injection (162 mg). Concomitant corticosteroid therapy was allowed. The study was classified as a category 3 (MR004) according to the French Jardé law and it was approved by the Ethics Committee of each of the 3 institutions.

### 2.3 Outcomes

Patients were evaluated at Day (D) 1 (the first day of treatment in the TCZ group; the first day requiring more than 6L/min of oxygen flow, in the non-TCZ group), D7, D14 and at the end of follow-up. Patients were followed from admission to the date of the last news or death. The primary endpoint was mortality. The secondary endpoints were: mortality at day D7 and D14, subsequent ICU admission (for patients not in ICU at baseline) and need of IMV, percentage of oxygen-free patients at D7, D14 and at the end of follow-up as well as biological parameters (lymphocyte count, C-reactive protein (CRP), ferritin) at D7 and D14.

### 2.4 Statistical analysis

Continuous variables were expressed as mean ± standard deviation (SD) or median with 95% confidence interval (CI). Categorical variables were expressed as numbers and percentages. Groups were compared using Mann-Whitney, Kruskal-Wallis or Friedman tests for continuous variables and Fisher's exact test for categorical variables. On the basis of the results of univariate analyses, variables with *P* value <0.20 were included in a stepwise multivariate logistic regression analysis in order to assess independent associations. The level of significance (*P* value) was set at .050 for all comparisons and analyses. Statistical analyses were performed using MedCalc software version 10.0.1.0 (Mariakerke, Belgium). The propensity score was performed using inverse probability of treatment weighting and double robust methods, further forcing several parameters already identified as death risk factors (age, sex, obesity, diabetes, steroids) in a large cohort of SARS-CoV2 infected patients (SAS-9.4 software, NC, USA).

## 3 Results

Baseline characteristics of the study population are presented in Table 1 and did not differ between TCZ and non-TCZ groups, including age, main comorbidities (hypertension, diabetes, obesity, chronic heart or lung disease) and infection severity at admission (respiratory rate, oxygen saturation in room air). Clinical and biological parameters (oxygen flow, lymphocyte count, CRP and ferritin serum levels) were also comparable between groups at D1. Of note, the rate of patients directly admitted into the ICU was 2-fold higher in the SOC group (44%) than in the TCZ group (22%) (*P* = .150). However, the rate of patients with invasive mechanical ventilation at admission was comparable (11% in the non-TCZ group; 18.5% in the TCZ group; *P* = .7). TCZ patients also received steroids more frequently (44% vs 7%; *P* = .002). The use of hydroxychloroquine (48% vs 33%), lopinavir/ritonavir (11% vs 26%) and the anticoagulation dosing were not statistically different between the TCZ and non-TCZ groups.

**Table 1:** Comparison of the features and outcome of patients treated with tocilizumab (TCZ) or without tocilizumab (non-TCZ).

Variables		All	TCZ (n = 27)	Non-TCZ (n = 27)	*P*
Baseline	Age (yr), median (95%CI)^#^	67.5 (59–71)	68 (57–74)	67 (53–71)	.658
	Men, n (%)^##^	46 (85)	23 (82.5)	23 (82.5)	1.000
	Main co-morbidities				
	- Obesity, n (%)	27/52 (52)	13 (48)	14/25 (56)	.592
	- Diabetes, n (%)	19 (35)	9 (33)	10 (37)	1.000
	- Hypertension, n (%)	38 (70)	21 (78)	17 (63)	.372
	- Pulmonary disease, n (%)	10 (18.5)	5 (18.5)	5 (18.5)	1.000
	- Cardiovascular disease, n (%)	8 (15)	4 (15)	4 (15)	1.000
	- **Tobacco (past/current), n (%)**	**17/46 (37)**	**4/22 (18)**	**13/24 (54)**	**.016**
	Clinical features				
	- Fever, n (%)	47 (87)	23 (85)	24 (89)	1.000
	- Dyspnea, n (%)	44 (81.5)	21 (78)	23 (85)	.728
	- Cough, n (%)	40 (74)	20 (74)	20 (74)	1.000
	- Anosmia, n (%)	7/46 (15)	3 (11)	4/19 (21)	.424
	- Dysgeusia, n (%)	6/46 (13)	4 (15)	2/19 (10.5)	1.000
	- Breath rate at emergency room, %	30 (25–33)	30 (24–36)	30 (24–36)	.958
	- SaO_2_ at emergency room (%)	91 (90–94)	90 (86–94)	93 (90–95)	.274
	- SaO_2_ at admission in the unit, %	96 (95–97)	96 (95–97)	95.5 (94–98)	.750
	- O_2_ flow at admission in the unit (L/min)	4.5 (4–6)	4 (3–8)	5 (3–10)	.910
	- CT-scan lung injury extent (27 vs 21), n (%)				.145
	<25%	7/48 (15)	4 (15)	3 (14)	
	25%–50%	18/48 (37,5)	7 (26)	11 (52)	
	>50%	23/48 (48)	16 (59)	7 (33)	
Day 1	Delay between symptom onset and hospital admission (d)	7 (5–7)	7 (5–8)	6.5 (4–7)	.789
	**Delay between symptom onset and aggravation (d)**	**8 (7–9)**	**9 (8–11)**	**8 (6.5–8.5)**	**.041**
	SaO_2_, %^∗^ (22 vs 24)	94.5 (93–95)	93.5 (92–95)	95 (93–98)	.173
	O_2_ flow (L/min)^∗^ (22 vs 24)	10 (8–13)	10 (6.6–15)	10 (6–15)	.578
	High flow, n (%)^∗^ (22 vs 24)	20 (37)	9/22 (41)	9/24 (37.5)	1.000
	Mechanical ventilation, n (%)	8 (15)	5 (18.5)	3 (11)	.704
	**ICU direct admission, n (%)**	**18 (33)**	**6 (22)**	**12 (44)**	**.151**
	C-reactive protein (mg/L)	125 (105–157)	181 (140–228)	162 (115–239)	.657
	Lymphocytes (/mm^3^)	870 (764–931)	860 (772–995)	870 (625–981)	.540
	Ferritin (ng/mL)	1465 (956–1971)	1852 (955–2166)	1386 (529–1877)	.240
Day 7	**C-reactive protein (mg/L)**	**23 (11–39)**	**10.7 (28–104)**	**52 (29–104)**	**<.001**
	Lymphocytes (/mm3)	1470 (1290–1790)	1550 (1290–2019)	1450 (753–1812)	.093
	Ferritin (ng/mL)	1107 (626–1350)	927 (573–1351)	1184.5 (793–1734)	.346
	ICU transfer, n (%)^£^	14 (39)	8/21 (38)	6/15 (40)	1.000
	Mechanical ventilation, n (%)^∗^	17 (37)	8/22 (36)	9/24 (37.5)	1.000
	Oxygen-free, n (%)	12 (22)	5 (18.5)	7 (25.9)	.745
	Death, n (%)	7 (13)	3 (11)	4 (15)	1.000
Day 14	**C-reactive protein (mg/L) (8 vs 4)**	**23 (3–91)**	**6 (1.9–48)**	**95**	**.027**
	**Lymphocytes (/mm**^**3**^**) (16 vs 10)**	**1405 (1261–1775)**	**1760 (1320–2131)**	**1260 (958–1406)**	**.027**
	Oxygen-free, n (%)	19 (35)	10 (37)	9 (33)	.785
	Death, n (%)	8 (15)	3 (11)	5 (18.5)	.477
EOF Outcome	Death, n (%)	11 (20)	5 (18.5)	6 (22.2)	1.000
	Oxygen-free, n (%)	29 (54)	12 (44)	17 (63)	.188
Concomitant treatment	Hydroxychloroquine, n (%)	22 (41)	13 (48)	9 (33)	.406
	Lopinavir/ritonavir, n (%)	10 (18.5)	3 (11)	7 (26)	.185
	**Corticosteroids, n (%)**	**14 (26)**	**12 (44)**	**2 (7)**	**.002**
	Anticoagulation				
	- Standard dose	22 (41)	9 (33)	13 (48)	.256
	- Intermediate dose	8 (15)	6 (22)	2 (7)	
	- Curative	22 (44)	12 (44)	12 (44)	

### 3.1 Effect of TCZ treatment

At D7 and D14, we found significantly lower levels of CRP in the TCZ group compared to the non-TCZ group, as expected (D7: 10.7 mg/L vs 52 mg/L; D14: 8 mg/L vs 95 mg/L, respectively) and a slight improvement of the lymphocyte count (D7: 1550/mm^3^ vs 1450/mm^3^; D14: 1760/mm^3^ vs 1260/mm^3^, respectively) (Table 1).

At D7, the rate of patients requiring ICU admission or invasive mechanical ventilation was comparable between groups (ICU admission: 38% vs 40%; invasive mechanical ventilation: 36% vs 37.5% in the TCZ and the non-TCZ group, respectively). There was no significant difference between groups, at D7 and D14, regarding the proportion of oxygen-free patients (18.5% vs 25.9% at D7 and 37% vs 33% at D14 in the TCZ and the non-TCZ group, respectively) and death (11% vs 15% at D7 and 11% vs 18.5% at D14 in the TCZ and the non-TCZ group, respectively). At the end of follow-up, both groups remained statistically similar regarding oxygen-free status (44% vs 63%) and death (18.5% vs 22%).

### 3.2 Factors associated with mortality

During the follow-up period (median of 28 days for the TCZ group, 21 days for the non-TCZ group), 11 patients (20%) died within a 4 days-median interval (95%CI = 2.8–20.1). When compared to the 43 live patients at the end of follow-up, in a univariate analysis (Table 2), patients who died were significantly elder (78 vs 64 years; *P* < 10^−3^); 82% of the deceased were older than 72 years, compared to only 23% of live patients (*P* < 10^−3^). The deceased patients were also more likely to have underlying chronic heart disease (36% vs 9%; *P* = .045) and lower lymphocyte count (540/mm^3^ vs 900/mm^3^; *P* = .050) at baseline. We did not find any significant difference regarding IMV (45.5% vs 46.5%), the use of hydroxychloroquine (45.5% vs 39.5%), lopinavir/ritonavir (18% vs 19%), corticosteroids (36% vs 23%), TCZ (36% vs 23%) or anticoagulation dosing between these 2 groups.

**Table 2:** Analysis of factors associated with SARS-CoV2 related death.

Variables		Deceased (n = 11)	Alive (n = 43)	*P*	OR (95%CI)	*P*
Baseline	Age (yr)^#^, median (95%CI)	78 (73–83)	64 (53–69)	<.001	1.15 (1.04–1.3)	.008
	Age > 72 yr, n (%)^##^	9 (82)	10 (23)	<.001	14.85 (2.7–80)	.002
	Men, n (%)	10 (91)	36 (84)	1.000		
	Main comorbidities					
	- Obesity, n (%)	6 (54.5)	21/41 (51)	1.000		
	- Diabetes, n (%)	5 (45.5)	14 (32.6)	.489		
	- Hypertension, n (%)	10 (91)	28 (65)	.144		
	- Pulmonary disease, n (%)	4 (36)	6 (14)	.185		
	- **Cardiovascular disease, n (%)**	**4 (36)**	**4 (9)**	**.045**		
	- Tobacco (past/current), n (%)	4/8 (50)	13/38 (34)	.443		
	- Tobacco (current), n (%)	1/8 (12.5)	3/34 (8)	1.000		
	Clinical features					
	- Fever, n (%)	9 (82)	38 (88)	.621		
	- Dyspnea, n (%)	10 (91)	34 (79)	.667		
	- Cough, n (%)	6 (54.5)	34 (79)	.129		
	- Anosmia, n (%)	0/8	7/38 (18)	.325		
	- Dysgeusia, n (%)	1/8 (12.5)	5/38 (13)	1.000		
	- Respiratory rate in the ER (/min)	26 (21–30)	32 (25–36)	.091		
	- SaO_2_ in the ER (%)	92 (87–95)	91 (89–94)	1.000		
	- SaO_2_ at admission in the unit (%)^∗^	95 (94–96)	96 (95–97)	.156		
	- O_2_ flow at admission in the unit (L/min)^∗^	10 (2–15)	4 (3–5)	.203		
	- CT-scan lung injury extent (10 vs 38), n (%)			.797		
	<25%	2 (20)	5 (13)			
	25%–50%	3 (30)	15 (39.5)			
	>50%	5 (50)	18 (47.4)			
	Delay between symptom onset and hospital admission (d)	5 (3–7)	7 (5–7)	.133		
	**Delay between symptom onset and aggravation (d)**	**6.5 (4–8.5)**	**9 (8–10)**	**.014**		
Day 1	SaO_2_, %^∗^ (10 vs 33)	92.5 (91–95)	95 (93–97)	.075		
	O_2_ flow (L/min)^∗^ (10 vs 33)	15 (7–17)	9 (6–12)	.059		
	High Flow, n (%)^∗^ (10 vs 33)	6/10 (60)	12/36 (33)	.163		
	Mechanical ventilation, n (%)	1 (9)	7 (16)	1.000		
	ICU direct admission, n (%)	4 (36)	14 (33)	1.000		
	C-reactive protein (mg/L)	188 (154–286)	164.5 (121–211)	.228		
	**Lymphocytes (/mm**^**3**^**)**	**540 (403–1084)**	**900 (820–960)**	**.050**		
	Ferritin (ng/mL)	962.5 (610–6086)	1618 (1133–2015)	.501		
Outcome and treatment	ICU admission, n (%)	5 (45.5)	27 (63)	.322		
	Mechanical ventilation, n (%)	5 (45.5)	20 (46.5)	1.000		
	Tocilizumab, n (%)	5 (45.5)	22 (51)	1.000		
	Hydroxychloroquine, n (%)	5 (45.5)	17 (39.5)	.743		
	Lopinavir/ritonavir, n (%)	2 (18)	8 (19)	1.000		
	Steroids, n (%)	4 (36)	10 (23)	.448		
	Anticoagulation, n (%)					
	- Standard dose	4 (36)	18 (42)	.709		
	- Intermediate dose	1 (9)	7 (16)			
	- Curative	6 (54.5)	18 (42)			

**Figure 1:**
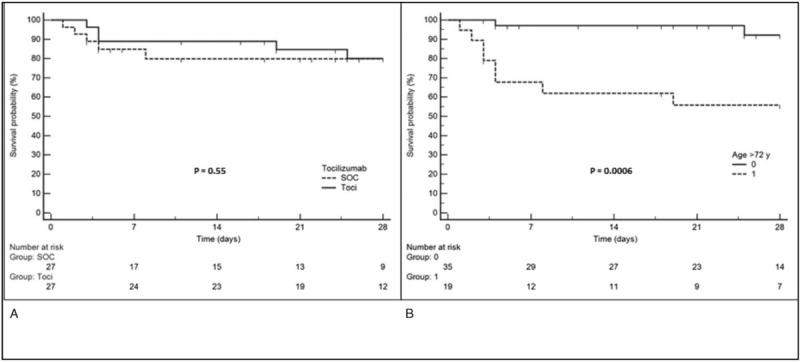
Kaplan-Meier survival curves: (A) tocilizumab (Toci) vs SOC; (B) age > 72 yr vs age ≤ 72 yr. SOC = standard of care group.

Using a stepwise logistic regression analysis, we found that age (odds ratio (OR) = 1.15, 95%CI, 1.04–1.3, *P* = .008) and age above 72 years (OR = 14.85, 95%CI = 2.7–80, *P* = .002) were the only independent variables associated with death (Fig. 1). Among the 5 TCZ-treated patients who died, the youngest patient was a 54-year-old obese patient (BMI 38 kg/m^2^), with chronic respiratory disease and ongoing tobacco consumption. The other 4 patients were 73, 77, 83 and 89-year-old. The 6 control-patients who died were aged between 71 and 84.

When the study was designed, the benefit of corticosteroids for COVID-19 management was debatable,^[9]^ but recently published Recovery^[11]^ and Solidarity^[10]^ trials brought enough proof of the efficacy of steroids (dexamethasone) in reducing COVID-19 mortality. Because the TCZ treated group more frequently received corticosteroids (44% vs 7%), we hypothesized that the potential efficacy of TCZ might have been tempered by a negative impact of corticosteroids on death rate. Thus, we generated a propensity score model using the balance diagnostic of inverse probability weighted treatment (IPWT) model (age, sex, obesity, diabetes and corticosteroids were used for adjustment). This IPWT logistic model did not ascertain a favorable effect of TCZ treatment on death occurrence (OR = 0.94, 95%CI = 0.8–1.12, *P* = .512), even after using a double robust method (OR = 0.89, 95%CI = 0.77–1.07, *P* = .142).

## 4 Discussion

The deleterious role of the cytokine storm accompanied by uncontrolled macrophage and monocyte cells activation and high levels of serum IL-6, interleukin 1 (IL-1) and Tumor Necrosis Factor (TNF) α was recognized early in the SARS-CoV2 epidemic.^[5,21]^ These data suggested that the use of cytokine inhibitors such as anti-IL-6 (tocilizumab, sarilumab) may reduce the risk of death in severe COVID-19 independently of ICU admission or mechanical ventilation.^[6,22]^ Our controlled study, comparing 2 well-matched groups with respect to comorbidities and risk factors for severe disease, shows no effect of TCZ as an add-on therapy to SOC for severe COVID-19 patients in reducing mortality or subsequent need for IMV, despite a better control of the inflammatory response.

**Figure 2:**
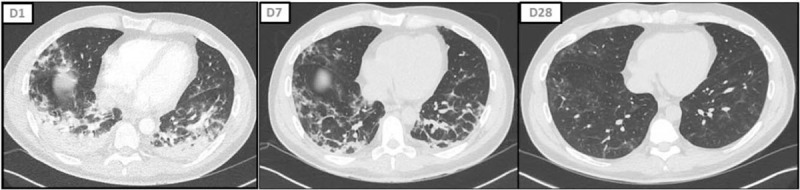
Chest computed tomography scan evolution of a 42-old patient treated with tocilizumab (intravenous, 600 mg).

Nevertheless, we cannot rule out that TCZ could be of use in some patients, as illustrated by Figure 2. Many authors came to the conclusion that the “anti-inflammatory treatment” should be initiated at the very start of the cytokine storm^[23]^ and some suggested that low doses of TCZ might prevent disease progression in patients with moderate COVID-19 and high inflammation.^[24]^ Some patients in our study may have thus received TCZ too late. Indeed, the interval between symptom onset and aggravation was 1 day longer in the TCZ group than in the non-TCZ group (9 vs 8 days respectively; *P* = .04). Two different patterns of immune dysfunction^[25]^ were suggested in SARS-CoV2-related ARDS; the first, driven by interleukine 1β (IL1β), suggestive of macrophage activation syndrome (hyperferritinemia and elevated H score for reactive hemophagocytic syndrome in up to 25% of patients); the second, driven by IL-6, consisting of immune dysregulation associating hypercytokinemia, immuneparalysis (as indicated by decreased Human Leukocyte Antigen – DR molecules on CD14 monocytes), and global lymphopenia. We hypothesize that the efficacy of anti-IL-1β (such as anakinra) or anti-IL6 (such as TCZ) molecules may be influenced by these patterns which may be present at different extents at the time the drug is initiated.

We focused our discussion on published manuscripts and excluded submitted pre-published papers. Several studies dealing with the use of TCZ have been published with different methodological designs (case-controlled or uncontrolled),^[13–16,26]^ but none were prospective or randomized. A metanalysis of 7 controlled studies showed no difference on death (relative risk (RR) = 0.61; 95%CI = 0.31–1.22) or ICU admission (RR = 1.51; 95%CI = 0.33–6.78) between TCZ treated groups and SOC groups.^[17]^ Another study, not included in the metanalysis, also failed to show a beneficial effect of TCZ on death or need for IMV; age equal or above 75 years was the only predictive factor of death in the TCZ patients.^[27]^

Very recent papers were published which showed a reduced risk of IMV and of death (adjusted hazard ratio (HR) = 0.61; 95%CI = 0.40–0.92; *P* = .020) in TCZ treated patients,^[18]^ a 45% decreased likelihood of death (HR = 0.55; 95%CI = 0.33, 0.90) despite higher superinfection occurrence (54% vs. 26%; *P* < .001) in patients under mechanical ventilation^[19]^ and decreased hospital-related mortality (HR = 0.64, 95%CI = 0.47–0.87; *P* = .0040) in TCZ treated ICU patients (HR = 0.64; 95%CI = 0.47–0.87; *P* = .0040)^[20]^ when compared to SOC. Another study found a non-significant tendency towards reduced mortality among ICU TCZ treated patients (HR = 0.76; 95%CI = 0.57–1.00)^[28]^ when compared with SOC patients. Finally, all awaited prospective randomized studies came just to be published, reporting the absence of TCZ efficacy in reducing COVID-19 mortality,^[29–33]^ thus confirming our first results.

In our study, TCZ was not associated with an improvement of the survival rate. The analysis of factors associated with death clearly indicated that older age was the main risk factor for death in all these reports, as we did. All other parameters including concomitant treatment with antibiotics, antiviral and presumably anti-inflammatory treatments were mostly non associated with death.^[34–36]^ Age above 72 years was the only independent factor associated with death, yielding a 15-fold superior risk. Indeed, when looking at the electronic health records of 17 million adult American patients, a 2-fold increase of the death risk was found in COVID-19 patients in the 60 to 70 year-old group, when compared with the 50 to 59 year-old group; this risk dramatically increased in patients older than over 80 years (HR = 12.64, 95%CI = 11.19–14.28).^[37]^ Noteworthy, male gender, grade II obesity and chronic respiratory disease such as chronic obstructive pulmonary disease were also associated with an increased risk of death in COVID-19.^[37]^ These 3 factors were the main features of the 54-year-old patient treated with TCZ who died in our study.

Despite 2 well-matched groups with respect to comorbidities and risk factors for severe disease, our study has several limitations including the retrospective, multicenter design as well as the limited number of included patients, which requires us to analyze and consider our results with caution. A center bias might be discussed as the 3 centers may have had different SOC. However, the baseline clinical parameters were comparable (age, medical history, sex, severity at admission). We can however note that patients included in one of the 3 participating hospitals were more severe than those of the 2 other centers. A time frame bias may be discussed. Indeed, 85% (23/27) of the control patients were recruited during March 2020 as compared to only 52% (14/27) of TCZ treated patients, whereas the SOC evolved between March and April 2020 with the better understanding of SARS-CoV2 infection. Also, in the propensity score analysis used to evaluate the impact of TCZ on death occurrence taking into account the higher proportion of corticosteroids treated patients in the TCZ group, TCZ failed to significantly influence death rate, but this should be considered with caution given the limited number of events (only 11 deaths).

In summary, despite an attractive and consistent rationale, supported by evidence of the association of increased serum IL-6 with severe SARS-CoV2 infection, our study failed to demonstrate a benefit of TCZ treatment for severe COVID-19 patients. Considering the limitations of our study and the discrepancies of new published results from well-matched or randomized controlled studies, the interest of TCZ treatment on death and ICU admission reduction is still debatable and deserves to be more clearly demonstrated.

## Acknowledgments

**Lariboisière COVID-19 group:** ALBERTINI Mathieu, AMADOR BORRERO Blanca, BOUAJILA Sara, BRITANY Kimbimbi, BURLACU Ruxandra, CACOUB Léa, CHAMPION Karine, CHAUVIN Anthony, DELCEY Véronique, DILLINGER Jean-Guillaume, FERON Florine, FRAZIER Aline, Thomas FUNCK-BRETANO, GALLAND Joris, GAUTHIER Diane-Cecile, GAUTIER Jean-François, HENRY Patrick, HUSCENOT Tessa, Sarah IZABEL Mathilde, JAULERRY, JOUABLI Moenes, JULLA Jean-Baptiste, KEVORKIAN Jean-Philippe, LALOI MICHELIN Marie, LEROY Pierre, LOPES Amanda, MANGIN Olivier, MEGARBANE Bruno, MICHON Maxime, MOULY Stephane, MUNIER Anne-Lise, NAHMANI Yoram, NICOL Martin, NICOLAS Eroan, POULAT Audrey, REVUE Eric, RICHETTE Pascal, RIVELINE Jean-Pierre, RUBENSTEIN Emma, SELLIER Pierre-Olivier, SENE Damien, THOREAU Benjamin, VODOVAR Dominique, ZANIN Adrien, AVENEAU Clément, BASTARD Paul, BEAUVAIS Diane, BOGHEZ Loredana, BORDERIOU Alix, CONWAY Paul, COSMA Lavinia, DAVY Vincent, DESJARDIN Clément, DEVATINE Sandra, DUCROZ GERARDIN Christel, DUPE Charlotte, GOBERT Chloé, GROS Clotilde, KADIRI Soumaya, KHAN Enmat, ONGNESSEK Sandrine, RHMARI Fatima, SACCO Isabelle, SAPTEFRAT Natalia, SCHAUPP Pauline, SERRE Justine, SICA Gabriel, SIDERIS Georgios, SMATI Sonia, TOURNIER Marine, TRECA Pauline, TRUONG Tony, TUFFIER Mathilde, ARCELLI Mattéo, BOUE Yvonnick, COPIE Alban, DEYE Nicolas, EKHERIAN Jean-Michel, ERRABIH, Zaccaria, GONDE Antoine, GRANT Caroline, GUERIN Emmanuelle, MAGALHAES Adèle, MALISSIN Isabelle, MEGARBANE Bruno, MEURISSE Edouard, MRAD Aymen, NAIM Giulia, NGUYEN Philippe, NITENBERG Kiyoko, PEPIN-LEHALLEUR Adrien, PERAULT Arthur, PERRIN Lucile, RENAUD Maxime, SUTTERLIN Laetitia, VOICU Sebastian.

**Diaconesses Croix Saint-Simon Hospital COVID-19 Group:** Thierry LAZARD, Benjamin SUBRAN, Karine MALEY, Pascal CHAZERAIN, Castille DECATHELINEAU, Grégoire SALTIEL, Arnaud VANJAK, Théo DHOTE, Yunyun MIAO, Louise GILLARD, Christiane STRAUSS, Nathalie LE GUYADER, Elisabeth KLEIN, Beate HEYM, Isabelle ETIENNEY, Jean-Michel DEVYS, Simon MARMOR, Roselyne DI MARCO, Isabelle VERBEKE, Aurélie DERVAUX, Françoise JULIEN, Nora CHOPFENBERG, Jeanine LAVIOLLE, Virginie CASTEL, Laurence MARSAL, Charles HOUDEVILLE, Vanessa POLIN, Axel EGAL, Elsa LAMBRESCAK, Thomas AUBERT, Antoine MOUTON, Florence AIM, Marie PROTAIS, Laure BERNARD, Andrea MECCARIELLO.

**Rothschild Hospital Foundation COVID-19 group:** Thomas SENÉ (Internist) and Pierre TROUILLER (ICU) would like to thank all the medical, paramedical and administrative staff involved in the management of the COVID-19 pandemic at the Rothschild Foundation Hospital.

All co-authors would like to thank all the medical, paramedical and administrative staffs involved in the management of the COVID-19 pandemic at Lariboisière Fernand Widal, Diaconesses Croix Saint-Simon and Rothschild Foundation Hospitals.

## Author contributions

**Conceptualization:** Ruxandra Burlacu, Jonathan London, Thomas Sene, Wladimir MAUHIN, Damien Sene.

**Data curation:** Ruxandra Burlacu, Jonathan London, Audrey Fleury, Thomas Sene, Vanina MEYSSONNIER, Valérie ZELLER, Joris GALLAND, Tessa Huscenot, Emma Rubenstein, Pierre Trouiller, Roland AMATHIEU, Johannes KUTTER, David BLONDEEL, Gabriel LEJOUR, Stephane Mouly, Olivier Lidove, Lariboisière Rothschild Diaconesses COVID19 Groups, Wladimir MAUHIN.

**Formal analysis:** Abdourahmane Diallo, Wladimir MAUHIN, Damien Sene.

**Investigation:** Joris GALLAND, Tessa Huscenot, Emma Rubenstein, Damien Sene.

**Methodology:** Ruxandra Burlacu, Abdourahmane Diallo, Valérie ZELLER, Emma Rubenstein, Wladimir MAUHIN, Damien Sene.

**Supervision:** Gabriel LEJOUR.

**Validation:** Jonathan London, Audrey Fleury, Olivier Lidove, Wladimir MAUHIN.

**Visualization:** Audrey Fleury, Roland AMATHIEU.

**Writing – original draft:** Ruxandra Burlacu, Jonathan London, Olivier Lidove, Lariboisière Rothschild Diaconesses COVID19 Groups, Damien Sene.

**Writing – review & editing:** Ruxandra Burlacu, Jonathan London, Audrey Fleury, Thomas Sene, Abdourahmane Diallo, Vanina MEYSSONNIER, Valérie ZELLER, Joris GALLAND, Tessa Huscenot, Emma Rubenstein, Pierre Trouiller, Roland AMATHIEU, Johannes KUTTER, David BLONDEEL, Gabriel LEJOUR, Stephane Mouly, Olivier Lidove, Lariboisière Rothschild Diaconesses COVID19 Groups, Wladimir MAUHIN, Damien Sene.
